# Gambling and hidden practices in forensic psychiatry: a qualitative study of ambivalence, autonomy, and negotiated care

**DOI:** 10.3389/fpsyt.2026.1758354

**Published:** 2026-03-11

**Authors:** Rasmus M. Hansen, Lisbeth U. Sørensen, Søren Kristiansen, Lisbeth Frostholm, Jakob W. Eriksen, Thomas Marcussen, Morten D. Terkildsen

**Affiliations:** 1 DEFACTUM - Social & Health Services and Labor Market, Central Denmark Region, Aarhus, Denmark; 2 Department of Forensic Psychiatry, Aarhus University Hospital, Aarhus, Denmark; 3 Department of Clinical Medicine, Health Aarhus University, Aarhus, Denmark; 4 Department of Sociology and Social Work, Aalborg University, Aalborg, Denmark; 5 Department of Functional Disorders, Aarhus University Hospital, Aarhus, Denmark; 6 Research Clinic for Gambling Disorder, Aarhus University Hospital, Aarhus, Denmark

**Keywords:** forensic psychiatry, gambling, qualitative, recovery, thematic analysis

## Abstract

**Introduction:**

This is the first qualitative study to examine how gambling becomes entangled with everyday life, relations, and recovery within medium-secure forensic psychiatric care in Denmark. The aim is to understand how gambling and financial management are experienced and understood by patients and staff, providing insight into how gambling practices intersect with ward life, clinical relations, and recovery processes.

**Methods:**

Semi-structured interviews were conducted with eight patients and seven staff members. The study followed an interpretive descriptive design and employed reflexive thematic analysis. Attention was given to participants’ accounts of gambling, money management, and the institutional conditions that shape these practices.

**Results:**

Patients described gambling as a way to manage boredom, relieve restlessness, and maintain a sense of agency, while also recognizing patterns of loss, debt, and relapse. Gambling was intertwined with attempts at self-regulation, familial involvement, and, at times, other addictions. Staff accounts were fragmented, ranging from viewing gambling as benign recreation to identifying it as a clinical concern linked to impulsivity, mood instability, or conflict. Across interviews, financial autonomy emerged as a central domain. Hidden economies, including informal loans and undisclosed debts, operated outside institutional visibility, complicating therapeutic relations and limiting staff capacity to intervene without eroding trust.

**Discussion and conclusion:**

Gambling emerged as an ordinary yet disruptive element of ward life, shaping autonomy and clinical relations and creating secrecy in relations. The discussion suggests that clearer expectations, routine enquiry, and responsive financial support could help address harm caused by gambling without undermining trust. Such measures may strengthen recovery processes, although the impact will depend on how they are integrated into the relational fabric of secure care.

## 1 Introduction

The organization of everyday life and care in a forensic psychiatric rehabilitation ward is shaped by the tension between care and control, autonomy and constraint (1–5). Within this structure, gambling may emerge as a practice that intersects with broader ward dynamics. Gambling studies, reveal how gambling behavior is linked to social, psychological, and financial harms, especially for vulnerable groups (6, 7).

Epidemiological studies reveal a broad continuum of gambling-related problems, ranging from low-risk gambling behavior to severe gambling disorder, with significant personal and social harms concentrated at the upper end of the spectrum (8, 9). Gambling disorder is classified in DSM-5 and ICD-11, and is defined by persistent and recurrent maladaptive gambling behavior that causes significant impairment and distress (10, 11). Research on gambling disorder has primarily focused on the general population or patients seeking treatment. However, vulnerable populations, like ethnic minorities, veterans, psychiatric patients, and prisoners, are at elevated risk, with prison populations showing a gambling problem prevalence of up to 30% (12–18). Nonetheless, research on gambling in forensic psychiatric settings remains limited. The development of gambling disorder is recognized as multifactorial (7). Epidemiological and clinical studies have repeatedly identified young, single, unemployed males with low educational attainment as being at high risk, a demographic profile overlapping with that of forensic psychiatric patients (7, 19, 20). Additionally, among people with gambling problems, there are associations with mood, anxiety, and other disorders including substance use and schizophrenia spectrum disorders that impact the prognosis, intervention, and clinical picture (21–23).

Furthermore, financial stress and debt have been linked to both criminal behavior and psychological distress, potentially impeding recovery and rehabilitation of forensic psychiatric patients (24, 25). As Kennedy & Davoran (26) emphasize, financial stability should be regarded as both a clinical outcome and a foundation for recovery in forensic psychiatric care. Recovery is typically described in terms of patient-centered components such as hope for the future, a sense of purpose, and empowerment (27–30). Yet these elements are challenging to sustain without basic economic security (31). Stable financial management enables patients to imagine a viable future, exercise responsibility, and sustain meaningful roles after discharge, thereby helping to promote the core function of forensic psychiatry of ensuring recovery while reducing the risk of recidivism. Consequently, financial stability is not peripheral but integral to recovery, and is identified as one of the seven essential “pillars of care and treatment” in forensic psychiatry (26).

As patients in forensic psychiatry are often admitted for more extended periods of time (32, 33), staff participation in patient interventions is deemed crucial, both in therapeutic administration and in influencing the circumstances of daily life within institutional settings (2, 3, 29, 30, 34, 35). Examining staff understandings of gambling behavior and its impact on treatment and ward life is thus critical to advancing knowledge in this area. While staff should be well-positioned to notice the presence of gambling and its consequences, little is known about how they detect and interpret these gambling practices, the strategies they employ in response, or the institutional rationales that shape their actions. However, focusing only on staff perspectives risks overlooking patients’ lived experiences, and recent studies increasingly argue that interventions should be guided by patients’ lived experiences to be meaningfully patient-centered (3, 5). However, currently, there exists a lack of empirical understanding regarding how forensic psychiatric patients and staff comprehend and navigate gambling practices such as debt handling and financial planning within forensic psychiatric settings.

This paper forms part of a larger study examining gambling among forensic psychiatric patients in Denmark. The associated quantitative paper (forthcoming) presents survey data from 214 patients across 19 forensic units, using the Problem Gambling Severity Index to compare gambling patterns with a gender- and age-matched national sample. The findings suggest that patients are about as likely to have gambled within the past year as individuals in the general population. However, among those patients who do gamble, the likelihood of experiencing gambling-related harm is considerably higher. In this context, gambling is therefore more likely to function as a sign of vulnerability than as a neutral recreational practice.

The present paper explores how gambling and financial management are experienced and understood by patients and staff within the everyday institutional context of a medium secure forensic psychiatric setting, thereby offering insight into how gambling practices intersect with ward life, clinical relations, and recovery processes.

We aimed to explore:

- How do forensic psychiatric patients perceive gambling and financial management?
- How do staff perceive and respond to these practices?
- What institutional conditions influence the management of gambling in secure psychiatric care?

## 2 Materials and methods

This study employed a qualitative design informed by interpretive description (36) and situated within an ethnographic approach (37, 38). Our analytical approach was thematic and was underpinned by a social constructivist orientation, drawing specifically on reflexive thematic analysis as an interpretive approach attentive to meaning-making, ambivalence, and complexity (39–41). This reflects our understanding that knowledge is constantly produced and interpreted within local contexts, where practices such as gambling, debt management, and economic negotiation are continually constructed by participants in relation to institutional structures, staff, and culture, and are embedded in narratives of autonomy, responsibility, trust, and risk. Rather than treating gambling and financial management as discrete behavioral phenomena, this approach attends to how they are rendered visible, regulated, and managed within forensic psychiatric care. This methodological approach, which combines visual and narrative gathering data with reflexive thematic analysis, allows themes to capture shared meanings, tensions, and ambivalences across accounts, without reducing experiences to predefined categories or variables, was chosen to highlight the often-overlooked, institutionally mediated practices of gambling and economic management in Danish forensic psychiatry. The aim is to create a nuanced account of how gambling, personal finance, agency, and treatment are experienced and negotiated within a forensic psychiatry ward.

### 2.1 Research team

The research team comprised of members with backgrounds in anthropology, sociology, psychology, and psychiatry, with experience spanning forensic mental health care, gambling research, and qualitative methods. Data collection and initial analysis were led by a qualitative researcher with no treatment authority at the ward, to reduce power asymmetries and facilitate open discussion of sensitive topics such as gambling and debt. At the same time, the team’s familiarity with forensic psychiatric practice and recovery-oriented frameworks informed analytic attention to institutional routines, relational dynamics, and ethical tensions. Reflexivity was practiced through ongoing discussion of how disciplinary assumptions, clinical knowledge, and normative commitments to recovery and patient autonomy shaped both the questions asked and the interpretations developed. The whole research team collaboratively designed the study protocol and regularly discussed analytic progress to guard against unexamined preconceptions.

### 2.2 Context

Interviews were conducted in six Danish medium-secure forensic psychiatric wards at two hospitals in Denmark. These sites represent distinct institutional spaces within the Danish context. Both institutions operate within the Danish welfare-state model and accommodate adult patients with diverse psychiatric, legal, and socioeconomic backgrounds. Close to 80% of the patients in medium forensic psychiatry are diagnosed with a psychotic disorder and often have comorbid substance use disorder. The majority of patients have committed severe person-related violent offences and have complex treatment needs, requiring a highly specialized and multi-treatment approach in a safe and secure setting. The wards are highly structured, with regulated routines and a degree of local variation in practices surrounding patient economy, personal autonomy, and daily life (29, 42). Patients in all the wards had access to personal digital devices and the internet during admission, subject to local rules and individual risk assessments. Online access formed part of everyday ward life and enabled activities such as communication with family members, social media use, gaming, and online gambling. Consequently, all patient accounts of gambling and financial practices reported in this study refer to experiences occurring during inpatient admission.

### 2.3 Participant recruitment and selection

We used purposive sampling to include participants with a range of experiences relevant to gambling and economic management (36). Recruitment and analytic focus were reviewed iteratively during data generation, guided by the concept of information power, where sample adequacy is assessed in relation to the specificity of the sample, the narrowness of the study aim, the theoretical anchoring of the analysis, the quality of interview dialogue, and the analytic strategy (43).

Eight patients and seven staff members were recruited from the six participating wards. All patient participants had completed the prior quantitative survey and were chosen to represent different levels of gambling involvement and economic vulnerability. As part of this survey, all patients completed the Problem Gambling Severity Index (PGSI). This validated self-report screening tool categorizes individuals who gambled within the previous year as non-problem, low-risk, moderate-risk, or problem gamblers (44). The selected patients represented all four PGSI categories. All patients who had reported gambling within the previous year were invited to participate in an interview and only one eligible problem gambling patient declined participation. All the patient quotes included are from interviews in which participants explicitly stated that they had experienced problems with gambling. Staff participants included nurses and social workers engaged in everyday patient support, including economic oversight. Participation was voluntary, based on written and oral informed consent. For the staff sample, one staff member from each of the six participating wards was interviewed, with an additional staff interview conducted in one ward to capture variation in local practices and perspectives.

Recruitment was concluded when the ongoing analysis indicated sufficient information power to address the study aim, namely, to develop an interpretive account of how gambling practices and money management are negotiated within everyday ward life and staff–patient relations (43). The resulting sample supports analytic transferability to comparable institutional contexts, while not warranting claims about prevalence or subgroup regularities.

### 2.4 Data generation

Data collection took place from October 2024 to February 2025 and was based on semi-structured interviews. The interview guide was informed by an ethnographic approach and lasted 25–50 minutes (37). It included open-ended questions about money management, forms and meanings of gambling, experiences of debt, and the perceived roles of staff and institutional routines in shaping these dynamics. For both patients and staff, the semi-structured format allowed for a combination of core research questions and openness to emergent themes and narrative elaboration.

To facilitate reflection and dialogue during patient interviews, we additionally drew on participatory design approaches (45) by incorporating visual card-based techniques developed to represent domains such as money, time, regular expenses, and gambling activities. These visual prompts, influenced by participatory and design-anthropological approaches, served as boundary objects, allowing participants to visualize financial routines, illustrate hidden aspects of economic life, and articulate practices associated with stigma or silence (46–49). This method encouraged participants to express tacit knowledge and exercise greater control over the direction and depth of the conversation.

### 2.5 Data analysis

Data were coded in NVivo 15 (50) and analyzed using reflexive thematic analysis (39–41), with theoretical inspiration from social science analyses of forensic psychiatric practice and recovery among forensic psychiatric patients in the healthcare system (3, 29, 51, 52). All interviews were transcribed verbatim prior to coding and analysis. Thematic development was advanced through collaborative interpretation within the research team. Analytic discussions were used to surface alternative interpretive possibilities, question implicit assumptions, and refine the developing themes considering the study’s social constructionist orientation. Reflexive thematic analysis treats the researcher’s interpretive engagement as an analytic resource (41). In this study, two researchers read the full dataset and met regularly to compare analytic observations, to deepen reflexive engagement with the data, explore tensions between patient and staff accounts, and strengthen the conceptual clarity of themes (41). We revisited patient and staff interviews during theme development to check that candidate themes captured patterned meaning across the dataset, while remaining attentive to variation and contradiction. Analytic weighting of the quotes was guided by their capacity to illuminate themes relevant to the study aim, rather than by an expectation of equal representational frequency across participants.

The themes presented were developed from all the interviews. In the presentation, some participants are quoted more extensively than others. This reflects differences in narrative elaboration and communicative ability, rather than differential analytic importance. In line with a reflexive thematic approach, longer extracts are used where they illuminate complex processes or tensions particularly clearly, while the analytic claims are grounded in patterns identified across the interviews rather than in any single case (41). Given the sensitivity of gambling and debt in a closed setting, we carefully considered the balance between analytic transparency and participant anonymity when deciding how to attribute extracts. Patients’ PGSI categories are thus not displayed to avoid identifying individuals on the wards.

Throughout the research process, the team actively addressed issues of positionality, trust-building, and the ethical challenges of representing vulnerable voices. These considerations were necessary given the participants’ institutionalized status and the sensitive, often stigmatized nature of gambling and financial struggles discussed in the interviews. The integration of visual methods and a reflexive analytic process ensured that participant perspectives shaped both the empirical material and the interpretative lens of the study, aligning with current participatory and patient-centered qualitative research (45, 48, 53, 54).

### 2.6 Ethics

The study adhered to the American Anthropological Association’s guidelines for ethical qualitative research (55). Ethical oversight was maintained in accordance with institutional and GDPR requirements. All participants gave informed consent and were reminded before each interview that they could withdraw at any time. Anonymity was maintained by carefully removing identifiable information from transcripts and reports. According to Danish law, no ethical committee approval was required for qualitative interview research without biological material (56). The study is registered at the Central Denmark Region List of Research (case no. 1-16-02-21-24).

## 3 Results

The findings are presented in two main sections that foreground patient perspectives and staff perspectives respectively. This structure was chosen to preserve the distinct experiential positions and asymmetrical responsibilities that shape how gambling and financial practices are understood within forensic psychiatric care.

### 3.1 Patient perspectives: navigating invisibility and everyday economies

#### 3.1.1 Living with self-regulation: cycles of hope and relapse

Patients described gambling as a recurring part of their everyday life. Experiences of gambling were marked by a cycle of attempted self-control, periods of abstinence, and eventual relapse, often tied to broader struggles with self-regulation. This dynamic was also evident in participants’ accounts of mobile gambling, where restraint was articulated as an ongoing effort. One participant explicitly framed her engagement in terms of trying rather than stopping: “*Yes, but I am trying to stop, because it is an expensive cost. I try not to play it, because it affects my phone bill.*” (Interview P.6). This emphasis on ‘trying’ situates self-regulation as provisional and fragile, a continuous practice rather than a stable achievement. The same participant described how this effort materialized through repetitive acts of deletion and return: “*Interviewer: How do you try to do that? P6: By deleting the game. Interviewer: So you delete it regularly? P6: Yes.*” (Interview P.6).

This ongoing attempt of “trying,” enacted through deletion and temporary abstinence, was also seen in how participants approached self-exclusion, where similar cycles of hope, restraint, and return were articulated through engagement with a formal system rather than through everyday app management alone. Several participants recounted periods of voluntary exclusion through Denmark’s ROFUS system (a national self-exclusion register that allows individuals to block their access to licensed gambling operators, both online and in physical venues, for a chosen period or permanently (57)). However, these acts of self-exclusion were understood as temporary, with patients describing a recurring tension between hope for self-control when gambling and the reality of repeated relapse.

As one patient put it, “I have actually been in ROFUS for a long time, and I just got out. I am a bit of a gambling addict, so now I am trying to see if I can take it fairly chill, where it does not run completely off track” (Interview P.1). At the same time, he explained how exclusion became a repeating cycle rather than a solution “I think I have been in ROFUS three times. It is silly. So far, it is going okay now. I have lost a little, but nothing serious. I like poker and sports betting. The problem is that sometimes I deposit 100 kroner just to win a bit extra. Then I lose, and it never comes back.” (Interview P.1).

Accounts of repeated self-exclusion underscored the iterative character of gambling behavior. Patients described a pattern in which small winnings or tolerable losses sustained optimism. Yet small deposits quickly became triggers for renewed gambling behavior. The act of “just” depositing 100 kroner illustrates the connection between intention and relapse, demonstrating how minor gambling bets could recalibrate the perceived risk of their gambling behavior.

For others, re-entry into ROFUS was planned in advance of the next downturn, a strategy that recognizes limited control while maintaining a sense of agency through timing. The planned return following a final payout reveals where the individual acknowledges incapacity while claiming choice.

“They say I should enroll in ROFUS. I cannot control it, and I will not be able to control it. This is where I have the choice. I am waiting for the money at Maria Casino to be paid out, and when they are used up, or whatever happens to them, then I will enroll in ROFUS again.” (Interview P.2).

This oscillation between withdrawal and re-engagement was further articulated by the same patient, who reflected on having actively opted out of self-exclusion in recent months: “I have done that over the past couple of months, because unfortunately I made the choice to withdraw from ROFUS again, because I thought I could control it” (Interview P.2).

As a result, gambling acts as an addiction in which patients constantly negotiate their daily practices. Across accounts, self-regulation was described as an ongoing calibration, through deletion, self-exclusion, reduced stakes, or planned withdrawal, where hope was repeatedly renewed and undone by small acts of re-engagement. Here, ROFUS served as a personal resolve, providing a sanctioned mechanism for pause without addressing the underlying cause of their gambling behavior.

#### 3.1.2 Boredom, restlessness, and the function of gambling

Patients linked gambling to the structure of ward life, describing how the monotony of institutional routines, especially in the evenings and at night, caused a sense of restlessness. Restrictions on leave and access to the gardens, along with a lack of meaningful activities, exacerbated feelings of restlessness.

For example, one patient emphasized that rules and confinement exacerbated restlessness. “You are not allowed to go out to smoke, and you are not allowed to walk in the corridor. I am very restless. I often pace up and down. When you have to sit for seven hours in your room with nothing to do, you cannot sleep. What are you supposed to do? Sometimes I would go in and gamble.” (Interview P.1). Here, gambling served as a practical response to impose restlessness, filling time when other forms of self-regulation are unavailable.

Gambling thus emerged as both a source of stimulation and an escape, a practice that temporarily alleviated emotional restlessness, marked the passage of time, and added meaning to otherwise unproductive hours. In addition, several patients made explicit comparisons to other forms of addiction, framing gambling as a substitute for former substance use.

For several patients, small bets provided a way to pass the time. “The problem is boredom, when you feel you have nothing to do, and then you might as well put 100 kroner on some match, so you have something to follow.” (Interview P.1). This framing of gambling as a response to boredom was echoed by other participants, who explicitly identified everyday monotony as a motivation for gambling. One patient directly linked gambling to boredom in daily life: “because I also see it as boredom.” (Interview P.2). When asked whether boredom was the reasoning for gambling, she replied, “Yes, exactly.” (Interview P.2). These examples demonstrate how gambling was used to pass the time within institutional routines and to cope with restlessness.

However, restlessness can be seen as more than just a situational reaction to institutional boredom. Restlessness is also a symptom commonly associated with gambling disorder. In this sense, gambling could alleviate, reproduce, and intensify restlessness, resulting in a self-reinforcing cycle of affective behavioral engagement. Within this dynamic, boredom serves as both an explanation and justification for continued gambling, blurring the distinction between coping strategy and symptom. This dynamic was further reflected by another participant, who located gambling squarely within moments of boredom: when asked why boredom was associated with gambling, he responded, “Because it is often when I am bored that I play.” (Interview P.6). Although he reported experiencing boredom only intermittently, he attributed this directly to the institutional environment, stating simply, “It is boring to be here.” (Interview P.6).

In addition, for some, gambling was linked to other addictions. One patient described how he previously used his weekly allowances to gamble and to fund drug use. Gambling here served as an enabling practice, a step in the substance dependence economy rather than an independent behavior pursued for its own inherent rewards. The patient reflected on how the two forms of dependency reinforced each other.

“I used my pocket money every week to gamble, at least a third of it, so I could win some money and afford drugs and splash out on cocaine. That was really why I gambled. It is two addictions on top of each other. And when I gambled, it was an addiction, so I had to stop.” (Interview P.3).

This illustrates how gambling can be used as both a substitute and a supplement. It provided stimulation in the face of boredom, but it was also integrated into a larger consumption pattern, increasing the risks of dependence and debt. Such experiences complicate the interpretation of gambling as a simple pastime or coping mechanism, instead of situating it within a complex addiction practice.

#### 3.1.3 Silence, secrecy, and hidden economies

Despite the importance of money in everyday life, patients described a communicative silence around economic issues. Money was frequently associated with autonomy, as well as shame, guilt, and a reluctance to involve staff. Financial difficulties, debts, and gambling-related issues were rarely discussed openly, keeping them hidden from institutional oversight.

One patient replied, when asked why he does not discuss his finances with others, *“*Because I think it is private, how much I have and how much I spend on different things.*”* (Interview P.4). Here, privacy serves as a protective boundary, separating finances from the realm of collective discussion. Similarly, another patient described the lack of financial conversations by stating: *“*We do not talk about money.*”* (Interview P.2).

This silence extended to relations with staff. When asked whether gambling practices were ever raised with employees, one patient simply responded *“*No.*”* (Interview P.5).

Behind the silence, patients described hidden gambling economies that circulated outside of the ward’s official oversight. Informal loans and debts, which were sometimes linked to external networks, posed threats of intimidation and violence. One participant described how failure to repay could result in threats and public shaming.

“Yes, but then you … the one I pay to, if I do not pay him, then he writes to a debt collector who comes out and who might beat you up and such. They are not nice people.” (Interview P.2).

These accounts demonstrated how silence and secrecy can coexist alongside complex, risky financial practices. Gambling and debt are not absent from institutional life, but can be hidden by stigma, fear, and a desire to preserve autonomy.

#### 3.1.4 Relational dimensions: gambling as a social practice

For some patients, gambling was not solely an individual pursuit but was embedded in relationships with family members, partners, and friends. In these cases, gambling became part of the social fabric within and beyond the institution, entangled with dependency on others and shared activity. Participation was described as a personal practice and as something sustained or facilitated by significant others, sometimes with their active involvement.

Gambling was described as a practice shared across external relations such as family members and partners while patients were admitted to the ward, facilitated through internet access. One patient explained how even during periods of self-exclusion he relied on his mother to place bets for him: *“*I have done that a few times, also when I was in ROFUS, where I got my mother to make a Euro Jackpot coupon or something.” (Interview P.1). Here, self-exclusion was circumvented through familial cooperation, suggesting that boundaries around gambling were negotiated relationally rather than enforced individually. Furthermore, he portrayed gambling with his father as a way to enjoy gambling without losing control: *“*It came about when I signed up for ROFUS. I actually like to gamble, but it just should not get out of hand. It does not when he has it.*”* (Interview P.1). The presence of a relative was framed as a form of containment, where the activity was legitimized by being placed under another’s oversight.

For others, gambling was entwined with intimate partnerships. One patient recounted how her partner did more than encouraged play by creating practical opportunities for her to gamble: *“*For example, my boyfriend, when he is cooking and cannot log out, then I play for him. He has also made an account for me.*”* (Interview P.5). Gambling in this account was not hidden from close relations but instead embedded in everyday domestic life, reinforcing its normalization and making abstinence more difficult.

These narratives demonstrated how gambling extended beyond the ward’s confines into wider relational networks. The involvement of family and partners blurred the boundaries between individual responsibility and collective participation, complicating both disclosure and intervention. Seeking help in such contexts risked implicating others, while continued gambling could also function as a means of maintaining connection and shared identity.

Taken together, the patient accounts depict gambling and money management as practices that are simultaneously ordinary, relational, and risky. Gambling functions as a means of coping with boredom, regulating affect, sustaining social ties, and asserting autonomy in a highly regulated environment, while also producing debt, secrecy, and vulnerability. These practices often remain hidden from staff, because they are embedded in domains that patients experience as private. As the following section shows, this invisibility shapes how staff come to recognize, interpret, and respond to gambling-related concerns.

### 3.2 Staff perspectives: uncertainty, fragmentation, and the boundaries of intervention

#### 3.2.1 Divergent understandings and institutional ambivalence

Staff described gambling in strikingly different ways, reflecting an absence of shared frameworks for how to understand and manage gambling within the wards. Some viewed gambling as a somewhat harmless pastime, comparable to other everyday activities that provided distraction or entertainment. Others, however, framed it as a clinical concern, particularly when it intersected with addictive behaviors or ward conflict. This divergence created an atmosphere of ambivalence, in which gambling was alternately trivialized, normalized, or problematized, depending on the staff member, ward, and situation.

One staff member expressed the lack of clarity directly, emphasizing the need for an agreed approach: “I think we need to figure out what the approach is, what the help is when someone gambles. Should it be equated with substance abuse, or should it not be equated with substance abuse? Someone has to decide that, I think. And not me. Then I can live with it if the thought was just: ‘Just let them gamble’.” (Interview S.1).

At other wards, gambling was not framed as a clinical concern but rather as an ordinary and even socially acceptable activity. Staff described how conversations about betting or gambling could be openly shared between patients and employees without restriction. One staff member explained: *“*No, not at all. It is not forbidden. And I actually think that many of the staff join in the conversations, because it is so common to do it. So, I actually think it is completely normalized to talk about. There are no rules here that they cannot gamble. You can also [laughs] dig yourself into an economic hole. It is really awful, but it is a right you have. And I also think that this openness makes a difference in that people also dare to say when there is a problem.*”* (Interview S.2).

Others positioned gambling as a minor or invisible activity compared to other challenges in the ward environment. One explained that, in their experience, patients did not gamble much, at least not visibly, and that it was often colleagues rather than patients who engaged with betting: *“*It is mostly colleagues, I would say. It is not patients, I think, who are into that. Not the patients. I think they are really good at being out in the environments, being active and playing some computer games, so it is in the form of PlayStation or that kind of game.*”* (Interview S.3).

In one of the wards, staff described how gambling had gradually come to be treated as a clinical concern, with practices changing over time. Where betting during supervised leave was once tolerated, it is now explicitly restricted and equated with substance misuse. This shift illustrates how, in that ward, awareness of gambling has been integrated into everyday routines. *“*Previously, when patients were on leave, if they wanted to go and place a bet, then we would just stand beside them. We do not do that anymore. Now we say: We equate that with misuse. You are not allowed to bet when you are on leave with us. You have to do that when you are on leave alone.*”* (Interview S.4).

These accounts illustrate that the clinical understanding of gambling is diverse among staff. Staff responses are fragmented, shaped by individual interpretation, personal thresholds for concern, and the ward’s approach. Based on staff accounts, gambling awareness is consistently absent from treatment plans and screening structures, leaving it without an established clinical place in the broader framework of care. This ambivalence signals both an uncertainty about the clinical significance of gambling and a lack of institutional guidance on how it should be addressed, leaving the issue suspended between being a regular part of everyday life and a potential therapeutic problem.

#### 3.2.2 Substitution, escalation, and clinical risk

Several staff members understood gambling as part of a broader continuum of addictive behavior, often emerging in the absence of substances. Gambling was described as one of the practices that filled the void left by alcohol or drugs, functioning as a substitute that provided stimulation, excitement, or relief. While staff sometimes portrayed this substitution as relatively benign compared to substance misuse, they also acknowledged the potential for escalation, especially when combined with impulsivity or periods of psychiatric instability.

One staff member explained: “To replace an addiction, which many of our patients had before they were admitted here. Then you replace it with something else, and that is cigarettes or sweets or something like that.” (Interview S.5). Gambling, in this framing, was one among several compensatory practices that sustained addictive dynamics even within the restrictions of the ward.

Others described gambling as carrying its own risks, particularly when amplified by psychiatric symptoms. One staff member connected gambling behavior to manic phases, noting how financial loss became entangled with impulsivity and mood instability: *“*When he is in his manic periods, then he gambles a lot. Then he spends a lot of his money, and again, it is this thing about him being very impulsive. And then he has difficulty controlling it. Another thing is that I also think it replaces an addiction. He used to misuse. Abstinent now because of the admission, of course. And then I think he misses - that it gives the same kind of - you get that quick fix, dopamine in the brain, maybe winning something and so on.*”* (Interview S.5).

In more severe cases, gambling was linked to direct clinical and relational risks. Escalation could trigger aggression, conflict, or paranoia, with debts becoming entangled in psychotic symptoms and distorted perceptions. One staff member noted: *“*It can be violent behavior. It can be threats. It can be assaults. It can be other things it triggers. The patient can become psychotic from it. Can get worsening or deterioration of symptoms. It may be that suddenly they have a completely different idea of what the patient actually owes than what has been lent. The patient may have been psychotic when they lent the money as well. So, it is a very clear picture that we do not do that here in the ward.*”* (Interview S.3).

Across these accounts, gambling was not treated as a uniform and constant problem but as a phenomenon that could shift from a seemingly harmless substitute to a destabilizing risk depending on context. Staff recognized both the continuity of addictive patterns and the potential for sudden escalation, yet their responses remained situated and case-specific, rather than guided by systematic institutional policy.

#### 3.2.3 Invisible economies and ethical dilemmas

Staff were acutely aware that financial transactions between patients often unfolded out of sight, creating what they referred to as “invisible economies”. These hidden exchanges included informal lending, debts, and repayment networks that extended beyond the ward into online and external relations. Because these practices rarely surfaced directly, staff had to rely on observation, fragments of disclosure, or indirect signs of distress. The lack of transparency raised ethical and clinical dilemmas, particularly when hidden debts worsened symptoms, heightened vulnerability, or posed safety risks.

One staff member described the difficulty of identifying gambling-related problems in the absence of open communication: “It is not something we catch out there, because what can you say - what I experience - there are no patients who say to us that they gamble or anything. I do not hear anything about it. And if they lose, it is not something they say to me either. It can just be a bad day that I might observe them having. That they might be agitated about something else.” (Interview S.3).

Another staff member recalled how a patient described cycling in and out of self-exclusion, but without seeking staff involvement. As they explained: *“*We have a patient … who says: ‘Well, for example, I have signed up for ROFUS several times, because then it gets out of hand, and I cannot control it’. But I think many of them have the thought: ‘I will solve this myself,’ instead of going to the staff and saying: ‘Help me with a solution here’.*”* (Interview S.2).

The sense that gambling and debt took place “in the shadows” was a recurring theme. Staff acknowledged that hidden transactions could escalate into conflict or coercion, yet they lacked concrete tools to intervene without breaching trust. As one put it: *“*Then we can of course react and act accordingly, but we cannot do anything in the hidden. We can only have a guess about it. But it does go on. I know that.*”* (Interview S.3).

Staff also reflected on why economic matters remained particularly sensitive to patients. One noted that money remained one of the few areas where patients could exercise autonomy in an otherwise highly regulated environment. As they explained: *“*You can feel that it is private and that people are a bit proud about it, because when you are admitted here … you are deprived of so much freedom. There are so many people who start deciding over you. So, the one thing, which is economy, which is such a big thing in life, that you want to hold on to, that you want to manage yourself … which I also understand.*”* (Interview S.5).

These reflections place financial privacy within the larger context of institutionalization, in which daily freedoms are restricted. The economy emerged as an issue of self-determination, which patients sought to defend against institutionalization. This caused persistent tension among the staff. The invisibility of gambling and debt limited their ability to intervene, but attempting to act without disclosure risked further eroding patient agency and weakening therapeutic alliances. Staff found themselves in a position, aware that hidden economies shaped ward life, yet unable to address them directly.

#### 3.2.4 Impact on therapeutic relations

Where gambling and financial management became visible, staff often found themselves positioned less as supporters and more as enforcers. Monitoring, restricting, or correcting behavior risked shifting the relational dynamic from collaboration to control, a shift that several staff described as straining the therapeutic alliance. Efforts to intervene were seen as necessary for safety and order, yet they also risked reinforcing secrecy and undermining trust.

One staff member reflected on how gambling could complicate everyday interactions and even limit opportunities for shared activity: *“*Yes, it has, but it is not great. It is not great to have to go around correcting all the time, so it has also affected the atmosphere. Also, the thing about - can you then sit and watch a handball match together? We have patients who are very passionate about football. Can you then talk about football and football results? It is also - how are they supposed to be there? Because of course you should be able to talk about your favorite team winning, or how it is going in the Champions League, or whatever - I do not know much about such things, right? But it is also a balance - how much can you talk about and how much can you not talk about? Because if that is one of the only things you can talk to a patient about, then we should not shut it down completely. We should still be able to talk with the patient about it.*”* (Interview S.4). The reflection highlights how gambling talk complicates everyday social life on the ward, as well as how difficult it is for staff to enforce a rule that patients should not discuss gambling, because repeated correction risks damaging the relation between staff and patients and undermining the possibility of ordinary and meaningful activities, such as watching sports together.

Another staff member underlined the difficulty of maintaining relationships when intervention became primarily disciplinary: “In relational work, it is not great to be the one who always has to say: ‘You must not, and you should’. We would rather collaborate with patients and be able to talk and be together properly about things, right? But if that behavior comes, where it is us and them against each other, then those fronts become sharp, and we do not want that. We do not. We have tried a lot to avoid it, but at least we had one patient where we had to be like that. Otherwise, we would rather collaborate and try to figure things out together.” (Interview S.1).

Taken together, these accounts reveal how gambling shaped therapeutic relations in subtle but significant ways. Staff were caught between supporting autonomy and preventing harm, often having to restrict behaviors that simultaneously functioned as sources of sociality and identity for patients. This balancing act underscored the fragility of trust in forensic psychiatric settings, where each intervention risks the fine line between care and control.

Across patient and staff accounts, gambling emerges as both pervasive and elusive, sustained by cycles of secrecy, ambivalence, and negotiation. For patients, it functions as a means of coping, passing time, and maintaining social bonds, while also reinforcing patterns of addiction and debt. For staff, it is a phenomenon that resists straightforward categorization, shifting between being seen as recreational, trivial, substitutive, or clinically significant, depending on context. These divergent perspectives reveal how gambling is entangled with broader questions of autonomy, privacy, and institutional authority. The findings point to the absence of a shared framework within the wards, leaving gambling suspended between visibility and invisibility, private choice and institutional concern. This unsettled status positions gambling as a site where patient agency and institutional governance repeatedly collide, shaping therapeutic relations and the lived experience of care.

## 4 Discussion

The analysis shows that gambling in Danish forensic psychiatry is part of an everyday practice that is entangled in interpersonal relations and addiction behavior. Patients described cycles of attempted control, brief abstinence, and relapse that were sustained by the monotony of ward life and by the affective payoffs of anticipation, risk, and temporary relief. Staff, in turn, portrayed gambling through heterogeneous lenses that ranged from benign pastime to proxy for addiction, which produced fragmented responses that were highly local and situational rather than guided by shared clinical frameworks.

The findings align with contemporary accounts of gambling harm as a continuum rather than a binary state. Small, apparently manageable bets that punctuate long evenings, or low-stakes wagers that render televised sport meaningful, can slide into loss spirals once impulsivity is primed, and access is immediate. Langham et al. (6) conceptualize harm across financial, relational, cultural, and health domains, and this multidimensional framing is visible here in how debts, shame, and secrecy travel with patients across the porous boundary between the ward and external networks. Staff narratives of sudden escalation during mood destabilization, and patient narratives of iterative self-exclusion, are consistent with clinical literature that positions gambling disorder alongside substance use disorders, highlighting shared features such as craving, impaired control, and reward dysregulation (7, 22). That some patients used gambling to try to fund or substitute other addictions underscores the clinical entanglement of behavioral and substance-related compulsions documented across epidemiological and treatment samples (7, 22).

The daily schedule for the wards helps to explain how these patterns emerge. Patients gambled during the unstructured periods of the institutional schedule, especially in the evenings and nights, when stimulation is scarce. This resonates with work on carceral settings where gambling operates as both coping and social currency, with elevated prevalence relative to the general population (12, 13). For the patients, gambling did more than fill time; it organized attention, provided topics for conversation, and, through small deposits and micro-wins, created a sense of agency otherwise attenuated by the strictures of secure care. Such practices, however, accrued hidden costs. Informal loans, external “collectors”, and the fear of reputational damage made disclosure hazardous. Silence around money, repeatedly emphasized by patients, served as a defense of what they see as one of the last domains of autonomy in an environment otherwise saturated with surveillance and rules.

In forensic psychiatry, clinical care is characterized by persistent tension between therapeutic aims and protecting the public, the organizations, and the patient from further harm, as well as the competing obligations to maintain patient privacy and ensure safety. Staff described their role as shifting between collaborative support and authoritative enforcement. When gambling was equated locally with misuse, staff felt compelled to monitor conversations, restrict leaves, or monitor spending. These moves may reduce immediate risk, yet they also risk fraying the therapeutic alliance, a cornerstone of effective intervention across modalities (35). Patient accounts of financial secrecy can be read, in part, as a form of protection of dignity and voice, both of which are central to recovery discourses in forensic settings (29, 34).

A central consequence of these findings is the need to integrate gambling treatment into a recovery-oriented framework that explicitly addresses financial security as an essential component of the therapeutic process. The literature on forensic recovery highlights hope, meaning, and empowerment as patient-defined anchors, while recent guidance in forensic services adds financial stability as a pillar of safe community reintegration (26, 29). The present findings show that money management is not an ancillary topic. It carries moral weight, structures risk and mediates trust. This emphasis on the ethical and relational dimensions of financial behavior also sets the stage for understanding how patients navigate formal control mechanisms. In this context, patients’ strategic use of Denmark’s ROFUS system illustrates agentic efforts to regulate access, creating a sense of safety and temporary refuge from gambling related harm for patients that can reduce anxiety and provide a feeling of stability, yet the cycling in and out of exclusion, and the ease with which family members can circumvent it, point to limits of individualized technological fixes without parallel relational and environmental support (58). Crucially, these practices unfolded during inpatient admission rather than being confined to periods of leave or pre-admission life. The availability of online access within the ward rendered gambling and its relational extensions an ordinary feature of everyday institutional life, rather than an external risk imported from the community. Family-mediated gambling therefore illustrates their permeability in a digitalized forensic care environment, where autonomy, restriction, and relational support are continuously negotiated.

The findings have several implications for practice. As a result, service development could benefit from proceeding on complementary fronts. Starting, there is a need for a shared understanding of what counts as gambling behavior in forensic psychiatry, since markedly divergent interpretations shape current practice. Building on such shared ground requires that gambling be addressed more systematically within everyday clinical work. Routine, non-stigmatizing enquiry about gambling and debt could increasingly be embedded into assessment, care planning, and review, thus potentially fulfilling a role similar to the way substance use, and suicidality are standard elements of risk formulation. Screening alone may be insufficient. Instead, problematic gambling behavior could benefit from being reserved as a more explicit topic in everyday forensic psychiatric care. By normalizing the topic, the threshold for disclosure could be lowered, paving the way for earlier, proportionate responses sensitive to patients’ needs and priorities (6, 7).

Our research reveals how gambling and forensic psychiatry intersect, generating specific challenges, dilemmas, and needs. We therefore argue that future research and development would benefit from clear, co-produced guidance that distinguishes recreational play from harmful patterns, specifies the status of gambling during escorted and unescorted leave, and sets out pathways for stepped support amenable to the conditions surrounding forensic psychiatry. As prior work on recovery in secure care demonstrates, interventions gain traction where they are experienced as collaborative, integrate staff and patients’ perspectives, and respect everyday meanings, rather than as unilateral controls that harden an us–them divide (3, 27, 29, 34). Additionally, a salient challenge requiring further work is the insight we found regarding the structural conditions that amplify boredom in forensic psychiatry. Lacking meaningful activities in outside hours was experienced as providing an engine for gambling behavior. Finding ways to counter such conditions thus requires further attention in research and development.

Ethical dilemmas around “invisible economies” require particular attention. Staff described knowing that lending, coercive debt collection, or off-ward financial ties were operative, yet lacking grounds to intervene without breaching privacy. This leads to a forensic predicament in which patient rights, third-party harms, and ward safety are simultaneously at stake. A potential response could involve developing transparent, pre-agreed boundaries. These agreements could include informing, at admission and at periodic intervals, about how the service understands financial exploitation and what triggers staff action. Recovery-oriented care relies on strong therapeutic alliances (28, 29), and care should be given to ensure that agreements are developed in ways that acknowledge the complexities of creating and sustaining staff-patients alliances and accommodate this project accordingly. This involves, where concerns arise, always attempting the least intrusive, alliance-preserving steps first, for example, an offer of budgeting support, facilitated contact with creditors, or time-limited spending plans co-signed by the patient. Where coercion or threats are suspected, safeguarding frameworks must be put in place. Such graduated practices are consistent with recovery-oriented care that neither abdicates risk management nor collapses into paternalism (29, 34).

The relational fabric of care remains decisive. Staff described how limiting everyday talk about gambling in communal areas can also constrict the informal conversations through which therapeutic rapport is often built. Staff and patients may turn to common topics such as sports. Still, the close association between sport and betting makes it challenging to determine when ordinary discussion becomes a potential trigger. Attempts to minimize exposure to cues, therefore, sit in tension with the need for casual, interest-based interactions that support the formation of therapeutic alliances, a dynamic also highlighted in relational studies of secure care. Decades of psychotherapy and nursing research converge on the finding that alliance, marked by agreement on goals and tasks and by a bond of trust, is a leading predictor of outcomes across diagnoses and settings (35). In secure psychiatry, where coercion is structurally present, alliance must be actively cultivated in the interstices of routine. Allowing conversation about gambling while setting clear limits on wagering during leave is not necessarily hypocrisy; rather, it should be understood as a calibrated attempt to hold space for identity, interest, and connection without colluding with harm, by balancing the Care-and-Custody-Dichotomy (59). Prior qualitative work shows that such relational calibration is precisely the terrain on which recovery is either enabled or foreclosed in forensic wards (2, 27, 34).

Several limitations temper the transferability of these conclusions. The sample is small, recruited from six Danish medium secure wards, and comprises participants willing to discuss sensitive financial matters. Hidden economies, by definition, escape full description, and accounts from those most enmeshed in coercive debt networks may be underrepresented. The study period captures a particular institutional rhythm and policy environment, which could shift with leadership changes or national guidance. Even so, the convergence of patient and staff narratives around invisibility, ambivalence, and negotiated practice suggests that the core processes described here are unlikely to be unique.

Future work should test integrated, patient-centered interventions that couple routine enquiry and brief psychoeducation with financial capability support, facilitated ROFUS enrolment and maintenance when desired, and purposeful expansion of non-wagering leisure. Comparative designs across wards with different local rules could clarify the effects of restrictive versus permissive regimes on disclosure, alliance, and harm. Mixed-methods studies that link qualitative accounts of hidden economies with administrative markers of incident reporting, debt advice uptake, or readmission would further illuminate pathways between ward practice and longer-term outcomes.

The data used in this study invites a reframing of gambling among forensic populations. Gambling in forensic psychiatry is not to be understood exclusively as a discrete risk of behavior that occasionally perturbs treatment, nor as a trivial pastime that belongs entirely to the private sphere. Forensic gambling is a complex, socially contextualized, and distributed, technologically mediated practice that crystallizes the central dilemmas of secure care, namely, how to sustain autonomy, protect from harm, and build alliances under conditions of constraint. Recovery-oriented services that take the financial and relational dimensions of everyday life seriously are better positioned to address those dilemmas with consistency and care.

## 5 Conclusion

This study provides the first qualitative account of gambling and financial practices in medium-security forensic psychiatry. The findings demonstrate that gambling and economic vulnerability can become integral to recovery, therapeutic relations, and the organization of everyday life in secure care. Although patients often maintain silence around money to preserve autonomy, this can allow hidden economies, coercive debt, and unrecognized harm to persist, while staff describe it through uncertain, fragmented perspectives shaped by competing institutional priorities.

A more transparent and consistent clinical approach to gambling in a forensic setting is needed. Future research and practice should focus on developing methods that support routine, non-stigmatizing enquiry about gambling and debt, establish clear expectations regarding acceptable and harmful gambling, and provide accessible financial support to enable early identification and intervention. Implementing these measures in a recovery-oriented manner can enhance patient autonomy while supporting safety, trust, and therapeutic alliance within forensic psychiatric care.

## Data Availability

Research data are not shared due to privacy or ethical restrictions. Requests to access the datasets should be directed to RH, ramhan@rm.dk.

## References

[1] Rahman OA Alexiou E Degl’Innocenti A Holmberg C Olaya-Contreras P . Forensic psychiatric care professionals’ experiences of communicating with foreign patients and their relatives: the importance of language and culture. Front Psychiatry. (2025) 16:3389/fpsyt.2025.1643806. doi: 10.3389/fpsyt.2025.1643806, PMID: 40852152 PMC12368502

[2] Terkildsen MD Kennedy H Jentz C Sørensen LU . Online video games and patient–staff power relations. A qualitative study of care and custody in forensic psychiatry. J Psychiatr Ment Health Nurs. (2021) 28:493–503. doi: 10.1111/jpm.12813, PMID: 34897910 PMC9306509

[3] Terkildsen MD Vestergaard LK Møllerhøj J Sørensen LU . Forensic psychiatric patients’ Perspectives on COVID-19 prevention measures: A qualitative study. J Forensic Psychol Res Pract. (2022) 24:245–67. doi: 10.1080/24732850.2022.2118095, PMID: 41783271

[4] Martin T Street AF . Exploring evidence of the therapeutic relationship in forensic psychiatric nursing. J Psychiatr Ment Health Nurs. (2003) 10:543–51. doi: 10.1046/j.1365-2850.2003.00656.x, PMID: 12956633

[5] Shetty SR Burke S Timmons D Kennedy HG Tuohy M Terkildsen MD . Patient perspective on observation methods used in seclusion room in an Irish forensic mental health setting: A qualitative study. J Psychiatr Ment Health Nurs. (2023) 31:393–404. doi: 10.1111/jpm.12979, PMID: 37929765

[6] Langham E Thorne H Browne M Donaldson P Rose J Rockloff M . Understanding gambling related harm: A proposed definition, conceptual framework, and taxonomy of harms. BMC Public Health. (2015) 16:1–23. doi: 10.1186/s12889-016-2747-0, PMID: 26818137 PMC4728872

[7] Potenza MN Balodis IM Derevensky J Grant JE Petry NM Verdejo-Garcia A . Gambling disorder. Nat Rev Dis Primers. (2019) 5. doi: 10.1038/s41572-019-0099-7, PMID: 31346179

[8] Cowlishaw S Merkouris SS Dowling NA Rodda S Suomi A Thomas SL . Locating gambling problems across a continuum of severity: Rasch analysis of the Quinte Longitudinal Study (QLS). Addictive Behav. (2019) 92:32–7. doi: 10.1016/j.addbeh.2018.12.016, PMID: 30579115

[9] Tran LT Wardle H Colledge-Frisby S Taylor S Lynch M Rehm J Degenhardt L . The Prevalence of Gambling and Problematic gambling: a Systematic Review and meta-analysis. Lancet Public Health. (2024) 9:e594–613. doi: 10.1016/s2468-2667(24)00126-9, PMID: 39025095

[10] American Psychiatric Association . Diagnostic and statistical manual of mental disorders. Diagn Stat Manual Ment Disord. (2013) 5. doi: 10.1176/appi.books.9780890425596, PMID: 41415524

[11] World Health Organization . International statistical classification of diseases and related health problems (11th ed.). WHO (2019). Available online at: https://icd.who.int/en/ (Accessed October 02, 2025).

[12] Adolphe A Khatib L van Golde C Gainsbury SM Blaszczynski A . Crime and gambling disorders: A systematic review. J Gambling Stud. (2018) 35:395–414. doi: 10.1007/s10899-018-9794-7, PMID: 30039276

[13] Tørdal EV Pallesen S Sagoe D Jones LØ Mahjabeen F . The prevalence of gambling problems in prison populations: A systematic review and meta-analysis. J Behav Addict. (2024) 13:25–35. doi: 10.1556/2006.2024.00005, PMID: 38459995 PMC10988396

[14] Grant JE Levine L Kim D Potenza MN . Impulse control disorders in adult psychiatric inpatients. Am J Psychiatry. (2005) 162:2184–8. doi: 10.1176/appi.ajp.162.11.2184, PMID: 16263865

[15] Cowlishaw S Hakes JK . Pathological and problem gambling in substance use treatment: Results from the National Epidemiologic Survey on Alcohol and Related Conditions (NESARC). Am J Addict. (2015) 24:467–74. doi: 10.1111/ajad.12242, PMID: 25950376

[16] Metcalf O Lawrence-Wood E Baur J Van Hooff M Forbes D O’Donnell M . Prevalence of gambling problems, help-seeking, and relationships with trauma in veterans. PloS One. (2022) 17:e0268346. doi: 10.1371/journal.pone.0268346, PMID: 35613121 PMC9132294

[17] Barry DT Stefanovics EA Desai RA Potenza MN . Gambling problem severity and psychiatric disorders among Hispanic and white adults: findings from a nationally representative sample. J Psychiatr Res. (2011) 45:404–11. doi: 10.1016/j.jpsychires.2010.07.010, PMID: 20800852 PMC3651857

[18] Banks J Waters J Andersson C Olive V . Prevalence of gambling disorder among prisoners: A systematic review. Int J Offender Ther Comp Criminol. (2019) 64:0306624X1986243. doi: 10.1177/0306624x19862430, PMID: 31315486

[19] Moreira D Azeredo A Dias P . Risk factors for gambling disorder: A systematic review. J Gambling Stud. (2023) 39:483–511. doi: 10.1007/s10899-023-10195-1, PMID: 36884150 PMC9994414

[20] Crocker AG Nicholls TL Seto MC Charette Y Côté G Caulet M . The national trajectory project of individuals found not criminally responsible on account of mental disorder in Canada. Part 2: the people behind the label. Can J Psychiatry. (2015) 60:106–16. doi: 10.1177/070674371506000305, PMID: 25886686 PMC4394710

[21] Vestergaard SV Ulrichsen SP Dahl CM Marcussen T Christiansen CF . Comorbidity, criminality, and costs of patients treated for gambling disorder in Denmark. J Gambling Stud. (2023) 39:1765–80. doi: 10.1007/s10899-023-10255-6, PMID: 37814135 PMC10627974

[22] Dowling NA Merkouris SS Lorains FK . Interventions for comorbid problem gambling and psychiatric disorders: Advancing a developing field of research. Addictive Behav. (2016) 58:21–30. doi: 10.1016/j.addbeh.2016.02.012, PMID: 26900888

[23] Lorains FK Cowlishaw S Thomas SA . Prevalence of comorbid disorders in problem and pathological gambling: systematic review and meta-analysis of population surveys. Addiction. (2011) 106:490–8. doi: 10.1111/j.1360-0443.2010.03300.x, PMID: 21210880

[24] Coid JW Kallis C Doyle M Shaw J Ullrich S . Identifying causal risk factors for violence among discharged patients. PloS One. (2015) 10:e0142493. doi: 10.1371/journal.pone.0142493, PMID: 26554711 PMC4640710

[25] Lind K Kääriäinen J Kuoppamäki S-M . From problem gambling to crime? Findings from the Finnish National Police Information System. J Gambling Issues. (2015) 30:98. doi: 10.4309/jgi.2015.30.10, PMID: 29777460

[26] Kennedy HG O’Neill C Flynn G Gill P Davoren M . Dangerousness understanding, recovery and urgency manual (the dundrum quartet) V1.0.30 Structured Professional Judgement Instruments for Admission Triage, Urgency, Treatment Completion and Recovery Assessments (2016). Available online at: https://www.academia.edu/125910702/DANGEROUSNESS_UNDERSTANDING_RECOVERY_and_URGENCY_MANUAL_THE_DUNDRUM_QUARTET_V1_0_26_01_08_13_Four_Structured_Professional_Judgement_Instruments_for_Admission_Triage_Urgency_Treatment_Completion_and_Recovery_Assessments (Accessed September 09, 2025).

[27] Marshall LA Adams EA . Building from the ground up: exploring forensic mental health staff’s relationships with patients. J Forensic Psychiatry Psychol. (2018) 29:744–61. doi: 10.1080/14789949.2018.1508486, PMID: 41783271

[28] Mann B Matias E Allen J . Recovery in forensic services: facing the challenge. Adv Psychiatr Treat. (2014) 20:pp.125–131. doi: 10.1192/apt.bp.113.011403, PMID: 38385431

[29] Clarke C Lumbard D Sambrook S Kerr K . What does recovery mean to a forensic mental health patient? A systematic review and narrative synthesis of the qualitative literature. J Forensic Psychiatry Psychol. (2015) 27:38–54. doi: 10.1080/14789949.2015.1102311, PMID: 41783271

[30] Evans C Deljavan S Zimmermann K Allen K Moghimi E Canning C . Determinants of readiness to implement forensic patient-oriented research: a study of barriers and facilitators in a high-secure hospital. Front Psychiatry. (2025) 15:3389/fpsyt.2024.1509946. doi: 10.3389/fpsyt.2024.1509946, PMID: 39882166 PMC11774836

[31] Langeland E Johnsen IF Sømme KK Morken AM Erevik EK Kolberg E . One size does not fit all. Should gambling loss limits be based on income? Front Psychiatry. (2022) 13:3389/fpsyt.2022.1005172. doi: 10.3389/fpsyt.2022.1005172, PMID: 36465287 PMC9709812

[32] Uhrskov Sørensen L Bengtson S Lund J Ibsen M Långström N . Mortality among male forensic and non-forensic psychiatric patients: matched cohort study of rates, predictors and causes-of-death. Nordic J Psychiatry. (2020) 74:489–96. doi: 10.1080/08039488.2020.1743753, PMID: 32248726

[33] Davoren M Byrne O O’Connell P O’Neill H O’Reilly K Kennedy HG . Factors affecting length of stay in forensic hospital setting: need for therapeutic security and course of admission. BMC Psychiatry. (2015) 15. doi: 10.1186/s12888-015-0686-4, PMID: 26597630 PMC4657210

[34] Mezey GC Kavuma M Turton P Demetriou A Wright C . Perceptions, experiences and meanings of recovery in forensic psychiatric patients. J Forensic Psychiatry Psychol. (2010) 21:683–96. doi: 10.1080/14789949.2010.489953, PMID: 41783271

[35] Horvath AO . The therapeutic relationship: From transference to alliance. J Clin Psychol. (2000) 56:163–73. doi: 10.1002/(SICI)1097-4679(200002)56:2<163::AID-JCLP3>3.0.CO;2-D 10718600

[36] Thorne S . Interpretive description. (New York: Routledge) (2016). doi: 10.4324/9781315545196, PMID:

[37] Spradley JP . The ethnographic interview. Australia; Belmont, Calif: Wadsworth Group/Thomson Learning (1979).

[38] Kvale S Brinkmann S . Interview: det kvalitative forskningsinterview som håndværk. København: Hans Reitzels Forlag (2015).

[39] Braun V Clarke V . Using thematic analysis in psychology. Qual Res Psychol. (2006) 3:77–101. doi: 10.1191/1478088706qp063oa, PMID: 32100154

[40] Braun V Clarke V . Reflecting on reflexive thematic analysis. Qual Res Sport Exercise Health. (2019) 11:589–97. doi: 10.1080/2159676X.2019.1628806, PMID: 41783271

[41] Braun V Clarke V . One Size Fits all? What Counts as Quality Practice in (reflexive) Thematic analysis? Qual Res Psychol. (2020) 18:328–52. doi: 10.1080/14780887.2020.1769238, PMID: 41783271

[42] Møllerhøj J . Offender recovery. Forensic patient perspectives on long-term personal recovery processes. Int J Environ Res Public Health. (2021) 18:6260. doi: 10.3390/ijerph18126260, PMID: 34207855 PMC8296032

[43] Malterud K Siersma VD Guassora AD . Sample size in qualitative interview studies: guided by information power. Qual Health Res. (2015) 26:1753–60. doi: 10.1177/1049732315617444, PMID: 26613970

[44] Ferris J Wynne H . The canadian problem gambling index. (Canada: Canadian Centre on Substance Abuse) (2001). Available online at: https://www.greo.ca/Modules/EvidenceCentre/files/Ferris%20et%20al(2001)The_Canadian_Problem_Gambling_Index.pdf (Accessed November 30, 2025).

[45] Brandt E Binder T Sanders E . Tools and techniques: Ways to engage telling, making and enacting. (2012), 145–81.

[46] Rowley J Jones R Vassiliou M Hanna S . Using card-based games to enhance the value of semi-structured interviews. Int J Market Res. (2012) 54:93–110. doi: 10.2501/ijmr-54-1-093-110

[47] Cornwall A . Question cards: Putting your cards on the table in the interview process. Qual Res. (2024) 25:777–82. doi: 10.1177/14687941241277749, PMID: 41783398

[48] Kjaersgaard MG Knutz E Markussen T . Design games as fieldwork: Re-visiting design games from a design anthropological perspective. Design Stud. (2021) 73:100994. doi: 10.1016/j.destud.2021.100994, PMID: 41788901

[49] Zhang Y Hennebry-Leung M . A review of using photo-elicitation interviews in qualitative education research. Int J Qual Methods. (2023) 22. doi: 10.1177/16094069231185456, PMID: 41783398

[50] Lumivero . NVivo. Lumivero (2025). Available online at: https://lumivero.com/products/nvivo/ (Accessed November 30, 2025). PMID:

[51] Hörberg U . Caring science and the development of forensic psychiatric caring. Perspect Psychiatr Care. (2014) 51:277–84. doi: 10.1111/ppc.12092, PMID: 25358488

[52] Askola R Nikkonen M Paavilainen E Soininen P Putkonen H Louheranta O . Forensic psychiatric patients’ Perspectives on their care: A narrative view. Perspect Psychiatr Care. (2016) 54:64–73. doi: 10.1111/ppc.12201, PMID: 27901274

[53] Sutton J Austin Z . Qualitative research: data collection, analysis, and management. Can J Hosp Pharm. (2015) 68:226–31. doi: 10.4212/cjhp.v68i3.1456, PMID: 26157184 PMC4485510

[54] Karnieli-Miller O Nissim G Goldberg M . ‘It’s in the cards’: the contribution of illustrated metaphor cards to exploring values within narratives. Qual Health Res. (2016) 27:138–51. doi: 10.1177/1049732315609897, PMID: 26489712

[55] American Anthropological Association . Ethical considerations when publishing fieldwork photos in online sources (2023). Available online at: http://www.ethics.americananthro.org/ (Accessed November 24, 2025).

[56] Indenrigs- og Sundhedsministeriet . LBK nr 1083 af 15/09/2017, Bekendtgørelse af lov om videnskabsetisk behandling af sundhedsvidenskabelige forskningsprojekter (2025). Available online at: https://www.retsinformation.dk/eli/lta/2017/1083 (Accessed November 21, 2025).

[57] Danish Gambling Authority . *ROFUS | Spillemyndigheden.* [online] spillemyndigheden.dk (2023). Available online at: https://www.spillemyndigheden.dk/en/rofus#-rofus- (Accessed November 30, 2025).

[58] Kraus L Loy JK Bickl AM Schwarzkopf L Volberg RA Rolando S . Self-exclusion from gambling: A toothless tiger? Front Psychiatry. (2022) 13:3389/fpsyt.2022.992309. doi: 10.3389/fpsyt.2022.992309, PMID: 36213894 PMC9537465

[59] Peternelj-Taylor CA Johnson R . Custody and caring: clinical placement of student: nurses in a forensic setting. Perspect Psychiatr Care. (2009) 32:23–9. doi: 10.1111/j.1744-6163.1996.tb00519.x, PMID: 9121864

